# Clinical characteristics of older Japanese patients with acute appendicitis: A post hoc analysis

**DOI:** 10.1002/jgf2.477

**Published:** 2021-06-30

**Authors:** Yosuke Sasaki, Fumiya Komatsu, Naoyasu Kashima, Tadashi Maeda, Yoshiko Honda, Nagato Shimada, Kimihiko Funahashi, Yoshihisa Urita

**Affiliations:** ^1^ Department of General Medicine and Emergency Care Toho University School of Medicine Ota‐ku Japan; ^2^ Department of General and Gastroenterological Surgery Toho University Omori Medical Center Ota‐ku Japan

**Keywords:** acute abdomen, appendicitis, C‐reactive protein, geriatrics, thrombocytopenia

## Abstract

**Background:**

Acute appendicitis (AA) in older patients can look different from AA in younger patients. Although it is crucial that primary care physicians can recognize AA in patients of any age, few Japanese studies have examined the characteristics of older AA patients. To address this, we evaluated the clinical characteristics of older Japanese patients with AA.

**Methods:**

We performed a post hoc analysis of the data from a previous Japanese single‐center study. We analyzed the clinical information of both younger (age: 16–64 years) and older patients (age: ≥65 years).

**Results:**

A cohort of 236 patients consisting of 219 (92.8%) younger patients and 17 (7.2%) older patients was evaluated. The median ages of the younger and older patients were 34 (interquartile range [IR], 24–45) and 78 years (IR, 74–81), respectively. The prevalence of complicated appendicitis (CA) (older: 41.2% vs. younger: 14.2%), comorbidities (70.6% vs. 13.2%), and thrombocytopenia (17.7% vs. 4.1%), along with serum C‐reactive protein (CRP) level (6.7 mg/dl vs. 1.0 mg/dl), was significantly higher in older patients. Significantly fewer older patients had epigastric pain (17.7% vs. 53.0%). Logistic regression evaluating the characteristics of older AA patients showed that CRP >5 mg/dl had a high odds ratio (OR) (5.01; 95% CI, 1.73–14.54), while epigastric pain had a low OR (0.24; 95% CI, 0.06–0.90).

**Conclusion:**

Our study reveals a higher prevalence of CA and comorbidities in older patients, and suggests that a lack of epigastric pain, thrombocytopenia, and higher serum CRP level are characteristics of older AA patients.

## Graphical abstract

To examine the characteristics of older patients with acute appendicitis, we performed a post hoc analysis of the data from a previous Japanese single‐center study analyzing the clinical information of both younger (age: 16–64 years) and older patients (age: ≥65 years) with acute appendicitis. Our study reveals a higher prevalence of complicated appendicitis and comorbidities in older patients and suggests that a lack of epigastric pain, thrombocytopenia, and higher serum CRP level (>5 mg/dl) are characteristics of older patients.

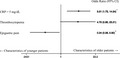

## 1 INTRODUCTION

Because older patients with acute appendicitis (AA) have higher mortality than younger patients due to their delayed diagnosis and higher perforation rate,^1^, ^2^, ^3^, ^4^, ^5^, ^6^, ^7^, ^8^, ^9^, ^10^, ^11^ primary care physicians must be able to recognize the distinct clinical presentation of AA in older patients in order to make a timely diagnosis. Previous studies, mainly from Western countries, have revealed that older patients tend to manifest atypical symptoms of AA: fewer than half of patients had the clinical presentations typically seen in younger patients.^6^, ^11^, ^12^ In Japan, however, studies on the characteristics and laboratory findings of older AA patients are sparse. To address this, we performed a post hoc analysis of the data from the previous single‐center study in Japan performed by Sasaki Y. *et al*.^13^ and evaluated the clinical characteristics and laboratory findings of older patients with AA.

## 2 METHODS

### 2.1 Design and patients

This study is a post hoc analysis of the data from previous Japanese single‐center study comparing the clinical characteristics of patients with simple and complicated appendicitis performed by Sasaki Y. *et al*.^13^ Here, we compared the clinical characteristics of younger adult patients (age: 16–64 years old) and older adult patients (age: ≥65 years old) who had been admitted to Toho University Medical Center Omori Hospital, a 948‐bed facility located in Tokyo, for treatment of AA between January 2012 and December 2016. The ethics committee of Toho University Medical Center Omori Hospital approved the study's protocol (M20130).

Because of the low incidence of appendectomy resulting from the “antibiotics first” management policy in place at the hospital, we included patients who had been diagnosed with AA by CT scan rather than by surgical findings. Patients were classified as having either simple appendicitis (SA) or complicated appendicitis (CA) based on CT and ultrasound findings as follows: Patients were diagnosed with SA if they had been clinically diagnosed with AA and had radiological/sonographical findings compatible with appendicitis catarrhalis or appendicitis phlegmonosa, such as swelling of the appendix or inflammatory changes of adjacent tissue, without findings suggesting CA. Patients were diagnosed with CA if they had gangrenous appendicitis, perforated appendicitis, or appendicitis complicated with an intra‐abdominal abscess. All CT and sonographic findings were reviewed by several different radiologists and surgeons within 48 h after testing.

### 2.2 Study variables

We collected clinical characteristics recorded at intake such as age, sex, time elapsed from the onset of symptoms to the time of the visit (onset‐to‐visit interval), epigastric/periumbilical pain, right lower quadrant (RLQ) pain, nausea/vomiting, diarrhea, anorexia, underlying diseases (e.g., diabetes, hypertension, hyperlipidemia, liver cirrhosis, hemodialysis, chronic lung diseases, renal dysfunction, and malignant tumors), immunosuppressant use, vital signs, RLQ tenderness, peritoneal signs, leukocyte count, serum sodium level, estimated glomerular filtration rate (eGFR), serum CRP level, and serum alanine aminotransferase (ALT) level, along with the CT and ultrasound findings at admission. We recorded symptoms and physical signs at the initial examination during the visit leading to admission.

### 2.3 Categorization of continuous variables

We categorized continuous variables according to the method reported in the previous study performed by Sasaki Y. *et al*.^13^ Fever was defined as an axillary measured body temperature of ≥38.0°C, and shock was defined as a systolic blood pressure <90 mmHg. Tachycardia was defined as a heart rate ≥100 beats/minute, and leukocytosis was defined as leukocyte count >11,000/mm^3^. Thrombocytopenia was defined as platelet count <150,000/mm^3^. Hyponatremia was defined as serum sodium <135 mEq/L, and elevated liver enzyme was defined as ALT >29 IU/L. Because it was difficult to distinguish acute kidney injury from chronic kidney disease in this retrospective study, we considered them together as renal dysfunction, defined as eGFR <60 ml/min/1.73 m^2^, and included this among the underlying disorders. CRP level was categorized into the following two groups: 0.0–5.0 mg/dl and over 5 mg/dl.

### 2.4 Statistical analyses

We compared all evaluated patient characteristics of both younger and older patients. The chi‐squared test was used for all dichotomous/categorical variables, while the Wilcoxon rank‐sum test was used for continuous variables because of their skewed distributions. Although our sample size was too small for a robust logistic regression model, we also performed a logistic regression analysis to adjust for confounding factors. Significant factors in the univariate analyses were selected as explanatory variables of the logistic regression. We examined the variance inflation factors (VIF) to evaluate the multicollinearity of the regression models. We evaluated the accuracy of the regression models by receiver operating characteristic (ROC) analysis. We also calibrated the models using the Hosmer–Lemeshow (HL) goodness‐of‐fit test. All statistical analyses were performed using Stata/IC software (version 15.1; Stata Corp, USA). A *p*‐value <0.05 was considered statistically significant.

## 3 RESULTS

This post hoc study evaluated a total of 236 patients (219 [92.8%] younger patients and 17 [7.2%] older patients). All patients were discharged without death or long‐term sequelae. Because of the institution's conservative management policy, appendectomy was performed in only 31/219 (14.2%) and 3/17 (17.7%) younger and older patients, respectively (*p* = 0.693). The results regarding each of the factors we examined and our univariate comparisons are listed in Table 1. The median ages of the younger and older patients were 34 years (interquartile range [IQR], 24–45) and 78 years (IQR, 74–81), respectively. Univariate comparisons revealed that the older patient group had a higher prevalence of CA (older, 41.2%; younger, 14.2%; *p* = 0.004) and of underlying diseases such as hypertension, dyslipidemia, and chronic lung disease (older, 70.6%; younger, 13.2%; *p* < 0.001), renal dysfunction (older, 11.8%; younger, 1.8%; *p* = 0.012), and thrombocytopenia (older, 17.7%; younger, 4.1%; *p* = 0.014). Serum CRP level was also significantly higher in older patients (*p* < 0.001). Notably, the proportion of patients complaining of epigastric pain was significantly lower among older patients (older, 17.7%; younger, 53.0%; *p* = 0.005).

**TABLE 1:** Clinical characteristics of patients

Factors	All patients (*n* = 236)	Older (*n* = 17)	Younger (*n* = 219)	*p*‐value
Age (years)	35.5 [25–50.5]^a^	78 [74–81]^a^	34 [24–45]^a^	NA
Male sex	129 (54.7%)	8 (47.1%)	121 (55.3%)	0.513
Complicated appendicitis	38 (16.1%)	7 (41.2%)	31 (14.2%)	0.004*
Appendectomy	34 (14.4%)	3 (17.7%)	31 (14.2%)	0.693
Onset–visit interval (days)	1 [0–1]^a^	1 [0–1]^a^	1 [1–1]^a^	0.362
Epigastric pain	119 (50.4%)	3 (17.7%)	116 (53.0%)	0.005*
RLQ pain	171 (72.5%)	14 (82.4%)	157 (72.0%)	0.343
Nausea/vomiting	123 (52.1%)	10 (58.8%)	113 (51.6%)	0.566
Diarrhea	46 (19.5%)	2 (11.8%)	44 (20.1%)	0.404
Anorexia	64 (27.2%)	3 (17.7%)	61 (27.9%)	0.362
Underlying diseases	41 (17.4%)	12 (70.6%)	29 (13.2%)	<0.001*
Hypertension	24 (10.2%)	9 (52.9%)	15 (6.9%)	<0.001*
Dyslipidemia	21 (8.9%)	6 (35.3%)	15 (6.9%)	<0.001*
Diabetes	11 (4.7%)	1 (5.9%)	10 (4.6%)	0.804
Chronic lung diseases	2 (0.9%)	2 (11.8%)	0	<0.001*
Renal dysfunction	6 (2.5%)	2 (11.8%)	4 (1.8%)	0.012*
Cancer	1 (0.4%)	0	1 (0.5%)	0.78
Immunosuppressant use	1 (0.4%)	0	1 (0.5%)	0.78
Fever	34 (14.5%)	2 (11.8%)	32 (14.6%)	0.747
Shock	6 (2.5%)	0	6 (2.7%)	0.489
Tachycardia	18 (7.6%)	3 (17.7%)	15 (6.9%)	0.106
RLQ tenderness	230 (97.5%)	16 (94.1%)	214 (97.7%)	0.364
Peritoneal signs	137 (58.1%)	12 (70.6%)	125 (57.1%)	0.277
Leukocyte count (/10^3^ mm^3^)	12.6 [10.1–15.2]^a^	12.4 [9.7–14.1]^a^	12.6 [10.1–15.2]^a^	0.499
Leukocytosis	159 (67.3%)	10 (58.8%)	149 (68.0%)	0.435
Platelet count (/10^3^ mm^3^)	225 [187–258.5]^a^	201 [181–276]^a^	227 [188–258]^a^	0.280
Thrombocytopenia	12 (5.1%)	3 (17.7%)	9 (4.1%)	0.014*
Hyponatremia	9 (4.1%)	9 (4.1%)	0	0.394
CRP (mg/dL)	1.1 [0.2–4.1]^a^	6.7 [2.8–11.5]^a^	1.0 [0.2–3.8]^a^	<0.001*
ALT >29 (IU/L)	38 (16.1%)	3 (17.7%)	35 (16.0%)	0.857
LDH >250 (IU/L)	33 (14.0%)	4 (23.5%)	29 (13.2%)	0.239

Abbreviations: ALT, alanine aminotransferase; CRP, C‐reactive protein; eGFR, estimated glomerular filtration rate; LDH, lactate dehydrogenase; NA, non‐applicable; RLQ, right lower quadrant.

^a^ Interquartile ranges are indicated in square brackets.

* *p*‐value <0.05.

**FIGURE 1:**
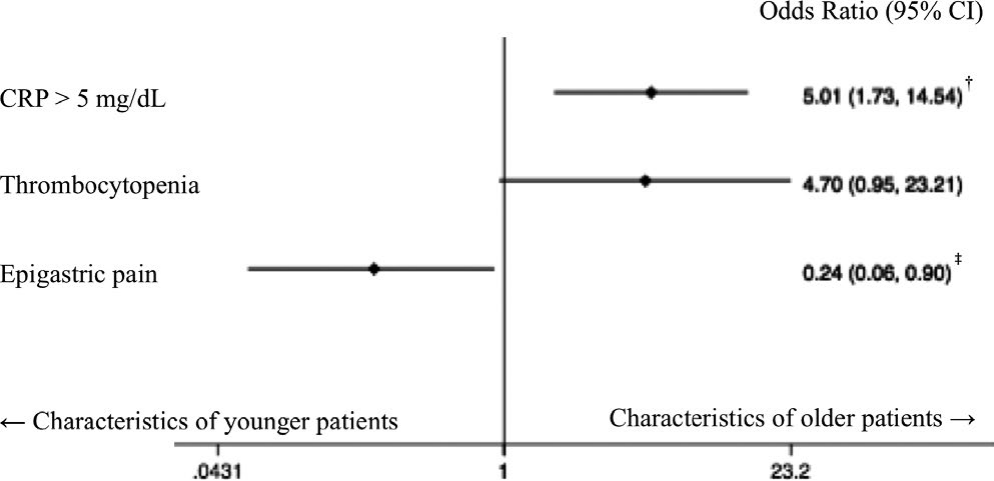
Characteristics of older patients with acute appendicitis (logistic regression). ^†^CRP >5 mg/dl has a significantly high odds ratio. ^‡^Epigastric pain has a significantly low odds ratio. CI, confidence interval; CRP, C‐reactive protein

Based on the result of univariate analyses, we performed logistic regression analysis to identify the clinical characteristics of older AA patients, which included epigastric pain, thrombocytopenia, and CRP >5 mg/dl as explanatory factors. The logistic regression model (Figure 1) showed that CRP>5 mg/dl had a significantly high odds ratio (OR): 5.01 (95% confidence interval [CI], 1.73–14.54; *p*‐value, 0.003). The presence of epigastric pain at the first visit had a low OR of 0.24 (95% CI, 0.06–0.90; *p*‐value, 0.035). Thrombocytopenia had a high OR of 4.70, although this was statistically insignificant (95% CI, 0.95–23.2; *p*‐value, 0.057). In this adjustment, we did not include underlying disorders as an explanatory variable because we interpreted the higher prevalence of underlying disorders among older patients with AA as simply a characteristic of the older population in general, not a hallmark of older patients with AA in particular. Similarly, we did not include complicated appendicitis as an explanatory variable because it was an outcome rather than a clinical feature that could be useful in diagnosis. The logistic regression model had moderate accuracy (area under the curve value of the ROC analysis: 0.758). The logistic regression model also had good calibration (HL chi‐squared: 4.19, *p*‐value = 0.242), and there was no multicollinearity (VIFs ≤1.03).

## 4 DISCUSSION

The present study on the clinical characteristics of older patients with AA revealed a higher prevalence of CA and underlying disorders, a lower prevalence of epigastric pain, a lower platelet count, and a higher serum CRP level in older AA patients. Each of these findings is significant.

The higher prevalence of CA in older AA patients is consistent with the results of the previous reports from Western countries. The higher proportion of perforated appendicitis in older AA patients is well known^1^ and is thought to be a cause of the higher mortality observed in such patients.^3^, ^5^, ^6^, ^7^, ^8^, ^9^ According to several review articles and previous reports, the proportion of perforated appendicitis and the mortality rate in older patients are 55–97%^7^, ^14^, ^15^ and 5–15%,^16^ respectively, in contrast to the respective values of 16–30%^15^ and <1% in younger patients.^16^ A higher prevalence of underlying disorders in older patients has also been previously reported.^7^ Although the possibility of an association between the higher rate of underlying disorders in older patients and their higher rate of mortality has not been explored, we think that the higher prevalence of underlying disorders is simply a characteristic of the older population in general, not a hallmark of older patients with acute appendicitis in particular.

The lower proportion of older patients who complained of epigastric pain, a noteworthy finding in the present study, has been proposed as an important cause of the delayed diagnosis of AA in older patients.^2^, ^7^, ^10^, ^14^ Because epigastric pain caused by elevated intraluminal pressure (localized RLQ pain is thought to be caused by subsequent localized peritonitis, i.e., CA)^13^ is thought to be a ubiquitous sign of early AA (i.e., SA), the higher prevalence of CA and the lower proportion of epigastric pain in our older AA patients are consistent with each other and with the previous hypothesis that the absence of epigastric pain, the “typical” sign of early AA, causes delayed diagnosis in the older population. Although our study did not show or evaluate differences in the prevalence of RLQ pain, RLQ tenderness, or migrating pain, a recent study has shown that AA patients aged >80 years had lower prevalence of migrating pain, higher prevalence of RLQ tenderness, and longer duration of symptoms.^11^ Considering that localized RLQ pain is thought to be caused by subsequent localized peritonitis following intra‐appendiceal pressure elevation, we think that this study supports the hypothesis that the absence of certain typical signs of early AA causes delayed diagnosis in the older population.

Similarly, we believe that the higher prevalence of CA in older patients can also explain their higher serum CRP level, given that high serum CRP level has been proposed as a parameter of CA, such as perforated appendicitis, in previous studies, including the original study performed by Sasaki Y. *et al*., in which the present data were gathered.^13^, ^17^, ^18^, ^19^, ^20^, ^21^ Regarding the significantly lower platelet count in older AA patients, we propose two possible explanations: First, thrombocytopenia as a manifestation of sepsis‐induced disseminated intravascular coagulation (DIC) may explain the higher prevalence of thrombocytopenia among older patients, as the prevalence of CA was higher and there were more cases with high serum CRP among our older patients. In this post hoc analysis, however, we could not evaluate fibrinolytic markers such as serum D‐dimer or fibrin/fibrinogen degeneration product to confirm this hypothesis. Second, age‐related changes in normal platelet count may explain the lower platelet count in older patients: A large study evaluating differences in platelet count associated with sex, ethnicity, and age in a normal population reported that platelet counts in participants aged 60–69 years and 70–90 years were lower by as much as 7,000/mm^3^ and 18,000/mm^3^, respectively, compared to the counts of younger participants (*p* < 0.001).^22^ Given that thrombocytopenia was statistically insignificant factor in the regression analysis, further investigation is warranted to determine the significance of the age‐related difference in platelet counts in patients with AA.

Our study has several limitations. First, due to the retrospective design of the original study, it lacks important information such as the precise length of the onset–visit interval in hours, the presence or absence of pain migration, Alvarado score,^23^ and respiration rate. In particular, the exact onset–visit interval in hours rather than days is highly relevant to the observed serum CRP levels because the time course after the onset of inflammation affects the serum CRP level: Serum CRP level rises rapidly for 4–8 h after the onset of inflammation and peaks at 48 h.^21^ Furthermore, because respiration rate is included in the diagnosis of sepsis,^24^ the lack of data on respiration rates may affect our assessment of thrombocytopenia associated with sepsis‐induced DIC. We also could not determine whether any renal dysfunction was due to acute renal injury associated with AA or whether it was merely a manifestation of underlying chronic kidney disease, because we did not have previous renal function data for all participants. Second, we lack pathological findings for the appendix because the surgeons use an aggressive strategy combining an “antibiotics first” approach with interval appendectomy as a form of advanced medical care.^25^ Third, our study has low statistical power due to its small sample size; we did not make a preparatory sample size calculation in this post hoc study using previously collected data; this may explain why our study did not detect some previously reported characteristics of older patients with AA such as longer onset–visit interval^7^, ^26^ and lower prevalence of fever^27^ as significant factors. We also note that the logistic regression model may not be statistically robust enough because of the small sample size. Nevertheless, we believe that our study can contribute to hypothesis generation because it does reveal some unreported characteristics such as higher serum CRP and lower platelet count. Further studies should include laboratory data and larger sample sizes.

In conclusion, this post hoc analysis evaluating the clinical characteristics of older patients with AA revealed that older AA patients have a higher prevalence of CA and underlying disorders, a lower prevalence of epigastric pain, lower platelet counts, and higher serum CRP levels. Our findings suggest that not only atypical clinical symptoms such as the lack of epigastric pain but also laboratory findings such as thrombocytopenia and higher serum CRP level can be characteristics of older patients with AA. Given the importance of this topic and the limitations of this study, further study is warranted.

## CONFLICT OF INTEREST

The authors have stated explicitly that there are no conflicts of interest in connection with this article.
